# Supplementary material for: Comparative genomics provides new insights into the diversity, physiology, and sexuality of the only industrially exploited tremellomycete: Phaffia rhodozyma
Source: BMC Genomics. 2016 Nov 9;17:901. doi: 10.1186/s12864-016-3244-7 (PMC5103461; doi:10.1186/s12864-016-3244-7)
Supplement: Additional file 6: — List of orphan genes with links to PFAM (related to Additional file 1: Table S1). (ZIP 1428 kb) [file 12864_2016_3244_MOESM6_ESM.zip › BLAST_HTML_FTR/G00898_P.html]

BLAST Search Results


```
BLASTP 2.2.27+


Reference:
Stephen F. Altschul, Thomas L. Madden, Alejandro A. Schäffer,
Jinghui Zhang, Zheng Zhang, Webb Miller, and David J. Lipman (1997),
"Gapped BLAST and PSI-BLAST: a new generation of protein database
search programs", Nucleic Acids Res. 25:3389-3402.


Reference for
composition-based statistics:
Alejandro A. Schäffer, L. Aravind, Thomas L. Madden, Sergei
Shavirin, John L. Spouge, Yuri I. Wolf, Eugene V. Koonin, and
Stephen F. Altschul (2001), "Improving the accuracy of PSI-BLAST
protein database searches with composition-based statistics and
other refinements", Nucleic Acids Res. 29:2994-3005.


Database: nr
           71,551,133 sequences; 26,053,659,533 total letters


Query= G00898_P

Length=436
                                                                      Score     E
Sequences producing significant alignments:                          (Bits)  Value

emb|CDZ98643.1|  hypothetical protein [Xanthophyllomyces dendrorh...   873    0.0  
ref|XP_008027590.1|  hypothetical protein SETTUDRAFT_91465 [Setos...  42.7    0.63 


 >emb|CDZ98643.1| hypothetical protein [Xanthophyllomyces dendrorhous]
Length=435

 Score =  873 bits (2255),  Expect = 0.0, Method: Compositional matrix adjust.
 Identities = 435/435 (100%), Positives = 435/435 (100%), Gaps = 0/435 (0%)

Query  1    MAAYHPRKVPPRPRSPPPTSSASSSHQNTDLLFPSAASAELAWSLVYPQRQRSVATSFSS  60
            MAAYHPRKVPPRPRSPPPTSSASSSHQNTDLLFPSAASAELAWSLVYPQRQRSVATSFSS
Sbjct  1    MAAYHPRKVPPRPRSPPPTSSASSSHQNTDLLFPSAASAELAWSLVYPQRQRSVATSFSS  60

Query  61   TTTTTSDTSSFAESPRVENRELSPSESAVPLPASYWHDLGLPAHDGHGNFPSPGITPRAS  120
            TTTTTSDTSSFAESPRVENRELSPSESAVPLPASYWHDLGLPAHDGHGNFPSPGITPRAS
Sbjct  61   TTTTTSDTSSFAESPRVENRELSPSESAVPLPASYWHDLGLPAHDGHGNFPSPGITPRAS  120

Query  121  IDGHLGFHRPETFHRSLSNRTADTYSTIDSVDDGLSALDLNDGSDYGWDGMESVRNRSEG  180
            IDGHLGFHRPETFHRSLSNRTADTYSTIDSVDDGLSALDLNDGSDYGWDGMESVRNRSEG
Sbjct  121  IDGHLGFHRPETFHRSLSNRTADTYSTIDSVDDGLSALDLNDGSDYGWDGMESVRNRSEG  180

Query  181  GSFGGGVAAEIGIGTGQTTGFTDRWRLSGEKRRRDSSPYTAVSVSTALSEEEEDQGRERE  240
            GSFGGGVAAEIGIGTGQTTGFTDRWRLSGEKRRRDSSPYTAVSVSTALSEEEEDQGRERE
Sbjct  181  GSFGGGVAAEIGIGTGQTTGFTDRWRLSGEKRRRDSSPYTAVSVSTALSEEEEDQGRERE  240

Query  241  QGGVHHHQQSSSRRISPTDLASTLYRRHSSLSSITLAGILTESSATPSSSPAGKAMTELL  300
            QGGVHHHQQSSSRRISPTDLASTLYRRHSSLSSITLAGILTESSATPSSSPAGKAMTELL
Sbjct  241  QGGVHHHQQSSSRRISPTDLASTLYRRHSSLSSITLAGILTESSATPSSSPAGKAMTELL  300

Query  301  TTSLGSLFQLASSSILSFLPPTELSYKHSIPLRVIRSVWNIDEDSLMLMFPPPLPRPVEQ  360
            TTSLGSLFQLASSSILSFLPPTELSYKHSIPLRVIRSVWNIDEDSLMLMFPPPLPRPVEQ
Sbjct  301  TTSLGSLFQLASSSILSFLPPTELSYKHSIPLRVIRSVWNIDEDSLMLMFPPPLPRPVEQ  360

Query  361  PVFHELFLKPISAPAIGPLEADKANEDGVKEAEGEAVLEASLEDGLNDHDDWQFESSNST  420
            PVFHELFLKPISAPAIGPLEADKANEDGVKEAEGEAVLEASLEDGLNDHDDWQFESSNST
Sbjct  361  PVFHELFLKPISAPAIGPLEADKANEDGVKEAEGEAVLEASLEDGLNDHDDWQFESSNST  420

Query  421  GQERMFRSPWMPWAG  435
            GQERMFRSPWMPWAG
Sbjct  421  GQERMFRSPWMPWAG  435


>ref|XP_008027590.1| hypothetical protein SETTUDRAFT_91465 [Setosphaeria turcica Et28A]
 gb|EOA85116.1| hypothetical protein SETTUDRAFT_91465 [Setosphaeria turcica Et28A]
Length=577

 Score = 42.7 bits (99),  Expect = 0.63, Method: Compositional matrix adjust.
 Identities = 42/158 (27%), Positives = 59/158 (37%), Gaps = 30/158 (19%)

Query  31   LLFPSAASAELAWSLVY-----PQRQRSVATSFSSTTTTTSDTSSFAESPRVENRELSPS  85
            +L P    +E AW L+      P  Q+S+        +    +  F E  +  +    P 
Sbjct  46   VLAPMVEQSEFAWRLLSRSFLPPSEQKSLLA-----YSPMFHSKMFGEKSQYRDSHFQPL  100

Query  86   ESAVPLPASYWHDLGLPAHDGHGNFPSPGITPRASIDGHLGFHRPETFHRSLSNRTADTY  145
            +SA+P P  Y+H   LP    H             +DGH  F RP T  +  SN   D  
Sbjct  101  KSALPSPVDYYHLSQLPDSSRH-------------LDGHPAFDRPLTV-QFCSNDPDDFL  146

Query  146  STIDSVDDGLSALDLNDGSDYGWDGMESVRNRSEGGSF  183
                 V     A+DLN G   G      +  R + GSF
Sbjct  147  RAAKHVAPFCDAVDLNLGCPQG------IAKRGKYGSF  178


Lambda      K        H        a         alpha
   0.312    0.128    0.380    0.792     4.96 

Gapped
Lambda      K        H        a         alpha    sigma
   0.267   0.0410    0.140     1.90     42.6     43.6 

Effective search space used: 4239809384382


  Database: nr
    Posted date:  Sep 23, 2015 12:05 AM
  Number of letters in database: 26,053,659,533
  Number of sequences in database:  71,551,133


Matrix: BLOSUM62
Gap Penalties: Existence: 11, Extension: 1
Neighboring words threshold: 11
Window for multiple hits: 40
```
